# Regional Heterogeneity in the Individual Unemployment Vulnerability After COVID-19 Outset

**DOI:** 10.1177/01600176231160486

**Published:** 2023-03-12

**Authors:** Ana Sofia Lopes, Ana Sargento

**Affiliations:** 1 CARME – Centre of Applied Research in Management and Economics, ESTG, 70866 Polytechnic of Leiria, Portugal

**Keywords:** job losses, COVID-19, regional effects, individual vulnerability, human capital

## Abstract

The COVID-19 crisis increased unemployment all over the World, with significant regional heterogeneity. This paper intends to analyze this territorial heterogeneity for the Portuguese case and investigate which regional factors complement personal and job characteristics in explaining individual vulnerability to COVID-19 unemployment. By considering personal, job and regional dimensions, we extended the literature and provided a more comprehensive understanding of this new phenomenon in the immediate and medium-term. Furthermore, this knowledge is essential to support policy suggestions for quick and effective action in preventing job losses in the current and future crises. Detailed information on all individuals that lost their jobs in Portugal 1 year after (and before) the COVID-19 outset was used to estimate three logit models that compare the odds of losing a job after and during the pandemic. Significant territorial heterogeneity of the COVID-19 impact on unemployment is obtained. Along with personal and job characteristics, we conclude that regional characteristics are essential for explaining individual vulnerabilities. In particular, workers are more prompted to lose their jobs if they live in regions with higher population densities, lower pre-crisis unemployment, and more dependable international flow. Conversely, individual and regional human capital investment contributes to protecting employment, revealing the existence of external effects.

## Introduction

One year after COVID-19 was declared a pandemic by the World Health Organization (WHO), more than 122.5 million people had been infected, and around 2.8 million died from this disease worldwide (WHO dashboard). Moreover, significant pressure was put on the health system, and the need to establish severe mitigation measures was raised. During 2020 and early 2021, several governments imposed emergency states and substantial restrictions on international mobility. Additionally, several economic activities not considered essential and impossible to perform remotely (from home) were closed. Thus, the COVID-19 pandemic caused severe economic impacts in addition to the devastating consequences on public health. The global GDP decreased by 3.3% in 2020, a value significantly higher than, for example, the 2008–2013 recession, which reported a peak of 1.3% reduction in 2009 (World Bank 2021). In Portugal, the GDP decrease was even higher, with a year-over-year (YOY) rate of −8.4% in 2020, while during the Great Recession, the peak was −4.1%. International trade was also severely damaged, and “from March 2020 to February 2021, compared with the previous 12 months, nominal exports (…) of goods registered rates of change of −11.1%” (INE 2021, 4).

As expected, a significant unemployment rate increase was also observed, reaching 7.2% and 6.5% in Portugal and worldwide, respectively (World Bank 2021). According to the OECD (2021), around 114 million jobs were lost worldwide in 2020. Considering the dimension of job losses and the associated socioeconomic consequences, studies investigating the impact of this unprecedented crisis on unemployment are essential for suggesting policies and practices aiming to protect jobs and the most vulnerable individuals.

Moreover, this crisis’s economic effects, particularly unemployment, were highly heterogeneous among countries and regions (OECD 2021). Thus, individuals who would be more protected from unemployment, considering exclusively their personal and job characteristics, may be more exposed to the crisis if they live in a more affected region due to external effects. Therefore, it is essential to understand which regional factors are behind these territorial differences and how they contribute to increased/decreased individual vulnerability.

The first goal of this study was to identify the heterogeneity of the economic effects (unemployment) of the COVID-19 crisis across the 23 NUTS III regions of Portugal (mainland). Second, we provided systematic evidence on how this regional heterogeneity affects the individual vulnerability to unemployment generated by the COVID-19 outset (compared with the pre-COVID-19 period). Third, we included an extensive set of personal and job characteristics to investigate if part of the regional effects on individual vulnerability is related to these characteristics. At the same time, we also analyzed how these individual characteristics affect the unemployment odds. Finally, by considering the literature on regional determinants and the particularities of the COVID-19 crisis, several key regional quantitative variables were introduced in the model, aiming to provide empirical evidence on how regional characteristics affect individual vulnerability in Portugal.

An extensive database that includes more than 800,000 individuals that lost their jobs in the observation period (between March 2019 and March 2021) was constructed by matching four different sources, explained in detail in a proper section. With detailed information in three dimensions (personal, job and regional characteristics), the data was then used to estimate three logit models where the odds of losing a job after the COVID-19 outset were compared with the unemployment odds before COVID-19.

Our analysis contributes to the literature on individual (un)employment determinants by adding regional to personal and job determinants. By working with micro-level data on personal and job’s characteristics, together with macroeconomic data referring to the region he/she lives, we provide a comprehensive analysis of how an economy-wide crisis affects a heterogenous population differently and control for the separate effect between the individual’s profile and the impact resulting from the regional context. Additionally, we help document the immediate and medium-term effects of the COVID-19 crisis, providing a broader understanding of this new phenomenon, which constitutes a valuable contribution from a theoretical point of view. Furthermore, this knowledge is essential to provide policy suggestions for quick and effective action in preventing job losses during crises.

The following section presents the theoretical background and our proposed contribution to the literature. The third describes the dataset and develops the modeling strategy. The results of the models’ estimations are presented and discussed in the fourth section. Finally, the main conclusions and policy/practical implications are drawn in the last section.

## Theoretical Background

The model specification and the correspondent explanatory variables were designed considering, firstly, the literature on (un)employment determinants at the individual level in a downturn. Within this specific theme, the previous economic recession (2008–2013) constituted a background for various empirical studies explaining the heterogeneous impact on unemployment through individual characteristics of workers, particularly gender, age, and education.

Concerning gender, the observed unemployment gender gap (the difference between female and male unemployment rates), which is commonly unfavorable to women, narrowed during the previous recession. This result was attributable to a higher increase in male unemployment rates, which, in turn, is explained by the fact that, in the most hard-hit sectors (e.g., construction and finance), the male workforce prevails (Passinhas and Proença 2020, for the Portuguese case; Cho and Newhouse 2013; De la Rica and Rebollo-Sanz 2017; García and Soest 2017). The recent studies on the COVID-19 impact unveiled an inverse gender result. Female workers mainly dominated the immediately affected sectors (predominantly tourism and hospitality), and the unbalanced responsibility (towards women) of family care during school closures, making women more vulnerable to unemployment (Adams-Prassl et al. 2020; Alon et al. 2020; Lopes, Sargento, and Carreira 2021; Montenovo et al. 2020).

Regarding human capital, previous empirical studies align with Becker’s (1962) theory which proposes that higher-educated workers have less unemployment vulnerability. This discrepancy is due to enhanced capabilities, skills and learning predisposition, a higher ability to adjust to changes and innovation and move from shrinking sectors to growing ones (Doran and Fingleton 2016; García and Soest 2017; Passinhas and Proença 2020). In the COVID-19 context, education level proved to be a resilience factor for individuals against crisis-provoked unemployment (Lopes, Sargento, and Carreira 2021, for the Portuguese context; Adams-Prassl et al. 2020; Couch, Fairlie, and Xu 2020; Daly, Buckman, and Seitelman 2020).

Besides individual characteristics, specific job/occupation features also determine vulnerability to unemployment. Indeed, the type of work contract is expected to affect unemployment odds, with temporary or more precarious employment bonds being related to a higher worker vulnerability (Bachmann et al. 2015; Baussola et al. 2015). This effect seems to also hold in the recent COVID-19 studies (Adams-Prassl et al. 2020; Fana, Pérez, and Fernández-Macías 2020; Kartseva and Kuznetsova 2020), although it was not corroborated by Lopes, Sargento, and Carreira (2021), who referred specifically to the hospitality sector. Additionally, and for the Portuguese case, Lopes, Sargento, and Carreira (2021) found statistical evidence for other job-related factors as explanatory variables of unemployment vulnerability. Supported by Becker’s (1962) reasoning on the relevance of job-specific skills protecting employment, the level of skills required by the previous occupation has positively impacted individual resilience within the hospitality context.

Finally, under the specific COVID-19 context, with the associated lockdown measures, working from home (WFH) feasibility has recently received increased attention. The rapid transition to remote work that occurred right after the pandemic outset allowed many jobs to be sustained and services to keep operating, thanks to the explosion of accessible and available web platforms and conferencing tools. However, occupations differ greatly regarding the share of tasks performed from a distance (i.e., WFH feasibility). Recently, Adams-Prassl et al. (2020), Beland, Brodeur, and Wright (2020), Fana, Pérez, and Fernández-Macías (2020), Lopes and Carreira (2022), and Sanchez et al. (2020) have included this factor in their analysis, concluding that occupations with a higher WFH feasibility are positively associated with the individual’s ability to avoid unemployment. Of course, such causality must control individual factors usually related to telework feasibility, such as higher skills or qualification requirements.

Most studies on (un)employment outcomes at the individual level only considered individual determinants. However, regional characteristics influence the whole regional labor market performance and, thus, indirectly, the odds of an individual becoming unemployed (Doran and Fingleton 2016; López-Bazo and Motellón 2013). Thus, considering the literature on regional determinants of crises’ impacts on regional economic outcomes (e.g., unemployment), a third group of determinants, region-specific factors, was included in the model. In selecting regional determinants included in the analysis, besides the common literature contributions, we also use variables that may assume particular relevance in the COVID-19 context.

Notably, the regional endowment in human capital has been identified as one of the most critical factors in reducing the impact of economic shocks. For example, an educated and skilled workforce enables the generation and absorption of knowledge and innovation in the region, which makes it better prepared to struggle against and adjust to economic shocks, particularly when combined with other regional competitiveness factors, such as high technology incorporation in production processes (Cappelli, Montobbio, and Morrison 2021; Crescenzi, Luca, and Milio 2016; Di Caro, 2017; Geelhoedt, Royuela, and Castells-Quintana 2021; Giannakis and Bruggeman 2017; Martin and Sunley 2015).

Until a certain threshold, population density is usually positively associated with better regional performance due to the agglomeration of economies that allegedly result from the urban concentration of people and firms (Geelhoedt, Royuela, and Castells-Quintana 2021). However, during disruptive and unexpected crises, “agglomerations may very well feel the adverse impact more when compared to other types of regions” (Palaskas et al. 2015, 6). In the particular context of COVID-19, this impact may be reinforced by the higher infection spread risk that densely populated regions face, with the potential consequences on restrictive mitigation measures and, thus, on the regional economy. Recent studies on the COVID-19 impact confirm that effect. Kartseva and Kuznetsova (2020) concluded that employees living in Russian regions with higher population densities are more exposed to unemployment risk. Additionally, Gong et al. (2020) included population density in their analysis and estimated that it had a negative short-term impact on the GDP growth rate of Chinese regions after the COVID-19 outset. In a recent study also applied to the Chinese context, Hu, Li, and Dong (2022) concluded that large cities with higher population density show higher vulnerability than small cities due to a higher risk of virus spread and, thus, heavier limitations on economic activity.

Pre-crisis unemployment has been used as an indicator of prior labor market performance. Although some studies establish a positive relationship between a priori unemployment rate and vulnerability to unemployment (Doran and Fingleton 2016), the majority of the literature concludes the opposite, i.e., regions performing better before the crisis, *ceteris paribus*, reveal higher unemployment vulnerability to the crisis (e.g., Cappelli, Montobbio, and Morrison 2021; Giannakis and Bruggeman 2017; Palaskas et al. 2015).

Given the disruptive nature of this crisis, some COVID-19-specific factors at the regional level might influence individual vulnerability to unemployment. The first refers to the share of employment in essential versus non-essential sectors. Herein, non-essential refers to forcefully closed activities under the lockdown and confinement measures applied throughout different countries because they did not satisfy fundamental needs. Although each government defined the list of activities obliged to close during the harshest lockdown period, these generally included, for instance, hospitality, restaurants, arts and leisure and some personal services. Some recent papers analyzed the impact of the share of employment in non-essential sectors and observed a negative impact on the regional labor market performance (Fana, Pérez, and Fernández-Macías 2020; Sanchez et al. 2020).

Also related to the industrial mix is the regional concentration in international dependent sectors: tourism and exports of goods. The constraints imposed on international mobility and the severe disruptions in global value chains have supported the inclusion of “international dependence” variables (such as weight of employment in tourism, economic openness degree or export propensity) as potential determinants of vulnerability to the COVID-19 crisis. Almeida and Santos (2020) for Portugal, Gong et al. (2020) and Hu, Li, and Dong (2022), in the case of China, concluded that the higher the share of international dependent sectors, the higher the vulnerability of the region to the COVID-19 impact.

Finally, the regional COVID-19 intensity may also affect individual vulnerability. According to Gong et al. (2020), regions with a higher incidence of infection might have more substantial effects on reduced consumption, firms’ closures and workers’ absenteeism, besides potentially implying the hardest confinement measures. Thus, the region’s economic impact will be more challenging, reflecting in more vulnerable workers.

**Figure 1.**
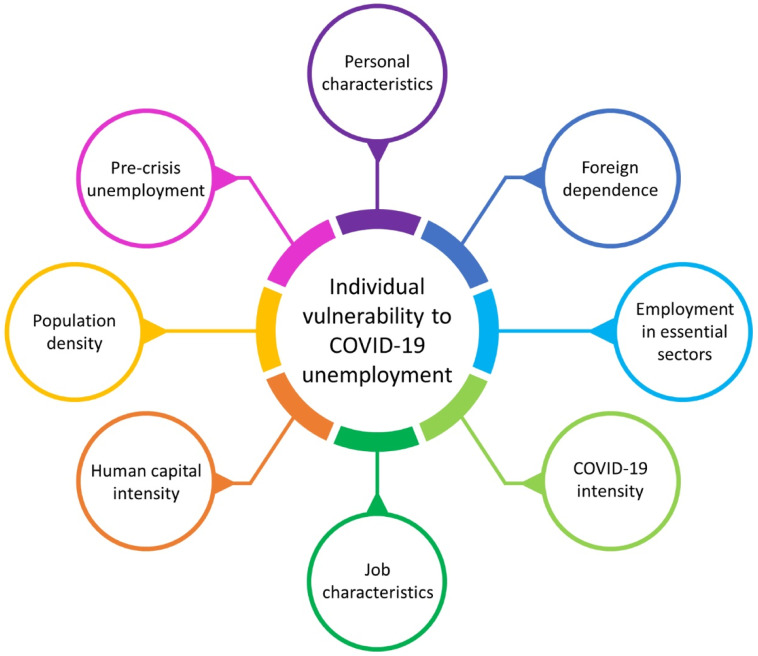
Determinants of individual vulnerability.

Herein, we combined and extended previous literature by considering a conceptual model, illustrated in Figure 1 below, that includes three dimensions that may influence the probability of an individual losing their job during a crisis: at the micro-level, their personal characteristics; at the meso-level, the characteristics of their job; and, at the regional level, the characteristics of the region where they live/work.

## Materials and Methods

Our database was built using information from the Portuguese Institute of Employment and Professional Training (IEFP) on all the individuals officially considered unemployed in Portugal. Since some characteristics of a previous job are also expected to influence the individual vulnerability to COVID-19 unemployment, we only consider individuals who had been employed before. Next, we selected two groups of observations: the pre-COVID-19 group (*Y*_1_), which includes all the individuals that lost their jobs during the year before COVID-19 hit Portugal (from March 2019 until February 2020); and the COVID-19 group (*Y*_2_) with individuals that had registered in the IEFP centers from March 2020 until February 2021 (during the COVID-19 crisis). Thus, our data includes the entire population of individuals who lost their jobs in Portugal during the observation period, corresponding to 801,798 people.

Following Lo (1986), we used a probabilistic regression model to perform a discriminant analysis between these two groups of observations. These models are standard for investigating employment/unemployment odds determinants (e.g., Dang and Nguyen 2021; Doran and Fingleton 2016; Lopes, Sargento, and Carreira 2021). The full set of observations is *Y*, with $Y_{1}\cup Y_{2}=Y$ and $Y_{1}⋂Y_{2}=0$. Since ${Prob}_{i}\left(Y_{2}|Y\right)$ = $1-{Prob}_{i}\left(Y_{1}|Y\right)$, the first logistic regression equation to be estimated will be

(1) $$\log\frac{{Prob}_{i}\left(Y_{2}|Y\right)}{{Prob}_{i}\left(Y_{1}|Y\right)}={\theta \tau }_{N\left(i\right)}+u_{i},$$

where the binary outcome variable is 0 for the observations in *Y*_1_ and 1 for those in *Y*_2_ (the COVID-19 group). The $\tau_{N\left(i\right)}$ is the set of dummies that identifies the region (at the NUTS III level), N, where the individual i lives and includes 22 NUTS III of mainland Portugal (the 23rd NUTS III, *Alto Alentejo*, is the baseline level in the regression model).^1^ The *ui* term denotes the error, and *θ* is an estimated parameter. A positive and statistically significant estimation for *θ* indicates that individuals living in the associated region are more vulnerable to unemployment after the COVID-19 outbreak than before COVID-19.

To investigate if the regional effects on individual vulnerability remain after controlling for several personal and job characteristics, the second equation to be estimated is an extension of equation (1) and includes micro-data information

(2) $$\log\frac{{Prob}_{i}\left(Y_{2}|Y\right)}{{Prob}_{i}\left(Y_{1}|Y\right)}={\theta \tau }_{N\left(i\right)}+\alpha X_{i}+\beta Z_{j\left(i\right)}+u_{i},$$

where X_i_ contains personal characteristics of individual *i* obtained by using information available at the IEFP database, including gender (with male = 1 and female = 0), age at the registration moment, the square of age, higher education (a dummy variable with 1 = yes, 0 = no) and a dummy identifying if it is the first registration at the IEFP centers of the individual *i*, or not.

Z_j(i)_ is a set of characteristics related to the last occupation, *j*, of individual *i*. For example, five dummies related to the reasons for being unemployed are considered: voluntary withdrawal from a permanent job, dismissed from a permanent job, laid off from a permanent job by mutual agreement (between employer and employee), end of a temporary contract, and loss of self-employment, (with other situations being the baseline level in the regression model).

Z_j(i)_ also includes some constructed variables obtained using the occupation code to match our data with external sources. First, the level of skills each occupation requires was identified using INE (2011) classification (the job-specific skills variables). The rank goes from performing simple and routine tasks included in the lowest skill level (Level 1–the baseline level in the model) to investigating specific domains and solving complex problems in the highest level (Level 4). Second, a variable that measures the WFH feasibility of the occupation was obtained by matching our data with the Martins (2020) database–WFH feasibility. For example, according to the classification of Martins (2020), a value of 0.67 for this variable indicates that around two-thirds of that occupation’s total labor hours can be performed from home. Finally, by comparing the previous occupation with the occupation that individuals intend to work in, we created and included a dummy signaling those who want to change to a different occupation. A detailed description of the variables and their summary statistics are provided in Appendix A.

These personal and job characteristics will, thus, be used to understand how they affect the probability of an individual being unemployed during the COVID-19 period (compared with pre-COVID-19) and to investigate if the regional effects are narrowed/disappear after controlling for these characteristics in the model.

Finally, several regional characteristics are introduced into the model to investigate their role in workers’ vulnerability to COVID-19 unemployment. Since IEFP data includes information on the residence county of each individual, it was possible to link our data with INE macro data on regional characteristics. Thus, our final model to be estimated includes a vector that contains a set of NUTS III (*N*) characteristics, R_N(i)_, and corresponds to

(3) $$\log\frac{{Prob}_{i}\left(Y_{2}|Y\right)}{{Prob}_{i}\left(Y_{1}|Y\right)}=\alpha X_{i}+\beta Z_{j\left(i\right)}+\gamma R_{N\left(i\right)}+u_{i},$$

R_N(i)_ includes the following variables:

- Pre-crisis unemployment measured by the percentage of unemployed individuals in the total population.
- Population density.
- Human capital intensity measured by the average number of schooling years of the employed population.
- Foreign dependence level evaluated by the percentage of the exports of goods in total turnover and the weight of the hospitality industry in total employment.
- Weight of the essential employment sectors. The list of essential sectors is presented in Appendix B and was obtained following the classification of Sanchez et al. (2020).
- COVID-19 intensity, measured by the month-average number of COVID-19 cases per 10 thousand inhabitants.

The model based on equation (3) includes personal, job and regional characteristics, which is expected to improve the explicative potential of the model regarding individual vulnerabilities to unemployment.

## Results and Discussion

### Regional Heterogeneity in Unemployment

**Figure 2.**
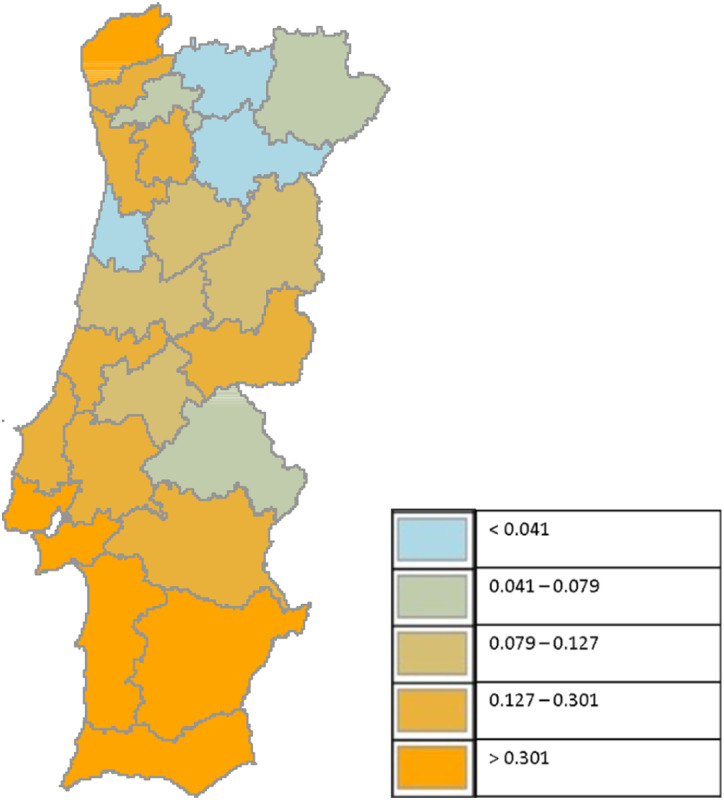
Territorial heterogeneity of the unemployment growth (variation rates).

Figure 2 presents the variation rates of the number of individuals registered in the IEFP centers of each of the 23 NUTS III in mainland Portugal between the pre-COVID-19 and COVID-19 periods.^2^ The regions were separated into five groups according to the level of unemployment variation. Consistent with our study, we only considered records from those employed before.

Figure 2 provides a simplified portrait of the ability of Portuguese regions to withstand the impact of a recessionary shock in the short/medium term. It also allows for preliminary identification of the most vulnerable regions to the COVID-19 impact on job loss. Substantial heterogeneity was observed between regions, which corroborates the findings of Almeida and Santos (2020) for the Portuguese regions. Some NUTS III present significant increases, above 30%, as in *Alentejo Litoral*, *Lisboa e Vale do Tejo* (LVT), *Algarve* and *Alto Minho*, the most hard-hit regions. In contrast, regions like *Alto Tamêga* and *Douro* contradict the COVID-19 impact on the unemployment trend and present a lower number of registrations the year after the COVID-19 outset than in the year before the pandemic.

### Models’ Estimations

In the first column of Table 1, we present the results obtained by estimating equation (1). Again, significant heterogeneity was observed among regions. The log-likelihood ratio of 2,496.00 (*p*-value = 0.0000) indicates an undoubted influence of regional effects on the probability of an individual being unemployed before and during the COVID-19 crisis. Regions with a positive and statistically significant coefficient seem to increase individual vulnerability to the COVID-19 crisis since the odds of being unemployed during the COVID-19 period compared to the pre-COVID-19 period is higher for individuals living in these regions.

**Table 1.** Logit Models (Regression Output, Odds Ratio).

	Variable	Model 1 (1)		Model 2 (2)		
*NUTS III*	Constant	0.0519	**	−0.3249	***	
	*Alto Minho*	0.2790	***	0.2934	***	+
	*Cávado*	0.1109	***	0.1776	***	+
	*Ave*	0.0228		0.0376		+
	*Grande Porto*	0.0793	***	0.1039	***	+
	*Alto Tâmega*	−0.0657	*	−0.0288		+
	*Tâmega e Sousa*	0.0993	***	0.1231	***	+
	*Douro*	−0.1121	***	−0.0354		+
	*Terras de Trás-os-Montes*	−0.0074		0.0177		+
	*Oeste*	0.1974	***	0.2020	***	+
	*Região de Aveiro*	−0.0299		0.0142		+
	*Região de Coimbra*	0.0742	***	0.1212	***	+
	*Região de Leiria*	0.1154	***	0.1500	***	+
	*Viseu Dão Lafões*	0.0733	***	0.1506	***	+
	*Beira Baixa*	0.0842	**	0.1469	***	+
	*Médio Tejo*	0.0355		0.0569	**	+
	*Beiras e Serra da Estrela*	0.0678	**	0.1574	***	+
	*Lisboa e Vale do Tejo*	0.2839	***	0.2787	***	−
	*Alentejo Litoral*	0.2925	***	0.2434	***	−
	*Baixo Alentejo*	0.2239	***	0.2190	***	−
	*Lezíria do Tejo*	0.0813	***	0.0771	***	−
	*Alentejo Central*	0.0873	***	0.0772	***	−
	*Algarve*	0.2778	***	0.2262	***	−
Personal and job characteristics	Gender (male)			0.0513	***	
	Age			0.0110	***	
	Age (squared)			−0.0002	***	
	Higher education			−0.0223	***	
	First unemployment			0.1439	***	
	Job-specific skills–level 2			−0.0159	***	
	Job-specific skills–level 3			−0.0544	***	
	Job-specific skills–level 4			−0.2554	***	
	WFH feasibility			−0.0826	***	
	Different occupation			−0.0845	***	
	Temporary job			0.3336	***	
	Permanent job (involuntary disruption)			0.4865	***	
	Permanent job (voluntary disruption)			−0.3516	***	
	Permanent job (mutual agreement)			0.1835	***	
	Self-employed			0.2978	***	
	Number of observations	801,798		801,798		
	Log-likelihood ratio	2,496.00(0.0000)		12,770.56(0.0000)		

*p-value <0.1, **p-value <0.05, ***p-value <0.01.

Compared to the residual region–*Alto Alentejo*–*Alentejo Litoral*, *LVT* and *Algarve* significantly impact the vulnerability of their residents to unemployment during this new crisis. This impact might be partially explained by the importance of the hospitality industry in the regional labor market. Indeed, these regions had the highest percentage of workers in this industry (25.6% in *Algarve*, 11.7% in *Alentejo Litoral* and 9.7% in *LVT*, 2017–2019 average). On the other hand, living in *Alto Tâmega* and *Douro* seems to facilitate individual permanence in employment during the COVID-19 crisis.

In column 2 of Table 1, a set of personal and job characteristics is included in the model–equation (2). As expected, individual characteristics are important in explaining workers’ vulnerabilities to COVID-19 unemployment (with the log-likelihood ratio increasing to 12,770.56). Additionally, we note that the Variance Inflation Factor (VIF) tests point to the inexistence of multicollinearity problems, with the natural exception of the variables age and age squared (VIF mean value of 3.23).

Concerning demographic characteristics, we observe that males, older workers, and those that do not have higher education degrees are more vulnerable to the unemployment generated by the COVID-19 crisis. The importance of human capital in protecting workers from unemployment in this new scenario is reinforced by the results observed for the job-specific skills. In fact, the higher the level of these skills, the more negative the coefficient is, which reveals that qualifications acquired during work also contribute to reducing unemployment vulnerabilities (Lopes, Sargento, and Carreira 2021).

Among the various reasons that led to registration in the IEFP centers, only voluntary disruptions are less prevalent after the COVID-19 outset, which might be related to individuals having lower expectations of finding a suitable job during the crisis. The other four reasons have positive coefficients. These results mean that the COVID-19 crisis also affects those traditionally more protected from unemployment. Interestingly, if only involuntary disruption is considered, the unemployment odds of workers with permanent contracts increase more from the pre-COVID-19 to the COVID-19 period than those with temporary contracts.

Workers in occupations with higher WFH feasibility are expected to be less vulnerable to COVID-19, considering the particular features of this crisis. The negative coefficient of WFH feasibility confirms this conclusion and completely agrees with previous literature (e.g., Adams-Prassl et al. 2020; Fana, Pérez, and Fernández-Macías 2020; Lopes, and Carreira, 2022; Sanchez et al., 2020). Considering the nature of this pandemic, we also analyze occupations’ requirement to be performed in proximity to other people. Thus, we use Faber, Ghisletta and Schmidheiny’s (2020) index to classify each job according to the physical proximity dependence and estimate an additional model that includes this variable. The coefficient of this variable is statistically significant and positive (0.0613), indicating that occupation’s dependence on physical proximity to co-workers or clients is also a vulnerability factor in this new crisis context.^3^

The most striking result emerging from equation (2) estimations is that those that desire to change from a different occupation are less vulnerable to the COVID-19 crisis than those that intend to maintain their current occupation. This result, combined with the positive coefficient of the first unemployment, highlights the disruptive nature of this crisis, generating new unemployment and driving workers away from their desired careers.

Introducing the individual characteristics–from equations (1) to (2) - reduces the regional impact differences in individual unemployment. The standard deviation between coefficients is smaller in column (2) of Table 1 compared to column (1). However, the territorial heterogeneity continues to impact the individual unemployment odds, even after controlling for individual characteristics. Thus, it is crucial to ascertain which variables, at the regional level, may contribute to a lesser or greater degree of vulnerability of their citizens.

In Table 2, we present the results obtained from the estimation of equation (3), which includes regional characteristics in addition to individual ones. We notice that the personal and job characteristics coefficients remain statistically significant and with the same signal as those obtained through the estimation of equation (2) (in column 2 of Table 1). Again, the VIF tests suggest the absence of multicollinearity problems (mean VIF of 2.63, after excluding the age square). The log-likelihood ratio is very similar to the one obtained in column 2 of Table 1, suggesting that the regional characteristics included in the model might be sufficient for explaining the effect of territorial heterogeneity on individual vulnerability.

**Table 2.** Logit Model (Regression Output, Odds Ratio).

	Variable	Model 3 (1)	
Personal and job characteristics	Constant	0.3932	***
	Gender (male)	0.0522	***
	Age	0.0110	***
	Age (squared)	−0.0002	***
	Higher education	−0.0218	**
	First unemployment	0.1458	***
	Job-specific skills–level 2	−0.0136	**
	Job-specific skills–level 3	−0.0541	***
	Job-specific skills–level 4	−0.2542	***
	WFH feasibility	−0.0801	***
	Different occupation	−0.0836	***
	Temporary job	0.3300	***
	Permanent job (involuntary disruption)	0.4836	***
	Permanent job (voluntary disruption)	−0.3502	***
	Permanent job (mutual agreement)	0.1808	***
	Self-employed	0.2974	***
Regional characteristics	Pre-crisis unemployment	−0.0674	***
	Population density	0.0002	***
	Human capital intensity	−0.0576	***
	Exports of goods	0.0017	**
	Employment in hospitality sector	0.0078	***
	Employment in essential sectors	0.0038	***
	COVID-19 intensity	0.0001	
	Number of observations	801,798	
	Log-likelihood ratio	12,318.64(0.0000)	

*p-value <0.1, **p-value <0.05, ***p-value <0.01

The negative and statistically significant coefficient of the regional pre-crisis unemployment suggests that individuals in regions with high pre-crisis performance in the employment outcomes are now more vulnerable to unemployment generated by COVID-19 and the associated mitigation measures. This result is interesting and reinforces the disruptive nature of COVID-19, as it affects more regions with traditionally lower unemployment. Cappelli, Montobbio, and Morrison (2021), Giannakis and Bruggeman (2017), and Palaskas et al. (2015) obtained similar results in the context of the Great Recession.

The higher the residence region’s population density, the higher the odds of individuals in these regions becoming unemployed during the COVID-19 period compared with the pre-COVID-19 period. This result is consistent with the studies of Giannakis and Bruggeman (2017), Gong et al. (2020), Kartseva and Kuznetsova (2020), and Palaskas et al. (2015).

The importance of the human capital level of the workforce is also in accordance with the literature. Moreover, our results indicate that, beyond the protection against unemployment for the individuals that invest in their human capital reinforcement, this investment also produces positive externalities, creating a regional environment that facilitates the regional adaptation to economic shocks (Giannakis and Bruggeman 2017) and reduces the odds of getting dismissed in this new context.

The positive coefficient of the importance of exports in total turnover indicates that individuals living in regions more reliant on foreign trade are more vulnerable to COVID-19 unemployment. This result is expected considering that the COVID-19 mitigation measures included the closure of land borders and international flight limitations, with severe disruptions in global value chains. The dependence on international flows is also related to the importance of the region’s tourism, namely the weight of the hospitality sector in total employment. Our study indicates that individuals who live in regions with higher employment weight in the hospitality industry are less protected from COVID-19 unemployment. Similar results were reported by Romão (2020), who shower several economic cycles in the 2006–2017 period, and Lopes, Sargento, and Carreira (2021), who reported on the present crisis in the Portuguese context.

Remarkably, the coefficient of the COVID-19 intensity has no statistical significance, which denotes that regions with a greater percentage of COVID-19 cases per inhabitant are not necessarily contributing to the increase in the odds of unemployment caused by COVID-19. This result may indicate that more than the narrowing of demand resulting from the risk of getting infected, unemployment may have been caused mainly by the mitigation measures taken by the government that may affect some regions more than others, depending on their economic structure. In fact, the majority and most severe measures to contain the disease were applied at a national level, in the period between March and June of 2020, which was also the period where the higher YOY variation rate of unemployment was observed, while the higher intensity of COVID-19 cases in Portugal occurred in the period from November 2020 until January 2021. Although most of the mitigation measures were equally established for the entire country, between June and December of 2020 some local and temporary sanitary measures were applied for territories with a relatively higher number of new cases per inhabitant. These measures included, for example, mandatory telework, stay-at-home duty and limited opening of commercial establishments and restaurants. Thus, to test for the conjecture of the mitigation measures’ impact on unemployment, a new dummy variable, identifying individuals that had lost their jobs in regions and during the period when special restrictions were applied, was created. When we replace the variable “COVID-19 intensity” with this dummy, this new explanatory variable has statistical significance and a positive coefficient (of 0.0539) without significantly changing the remaining coefficients. This result suggests that mitigation measures increase the vulnerability to unemployment of individuals who live in those affected areas during those periods. Our results seem to be consistent with Webster, Khorana, and Pastore (2021), which reported that the magnitude of the effect of government response in the permanent closure of firms in Southern Europe and consequently the loss of employment was significantly higher than the magnitude of the COVID-19 intensity effect.

Finally, the most surprising result is that individuals that live in regions where the essential sectors have a higher weight in regional employment are more vulnerable to COVID-19 unemployment. A deeper analysis of this result was made by estimating a model where only the lockdown period (between March and May of 2020) and the homologous period were considered. For these particular periods, the effect of being in a region with a higher weight of the essential sectors is negative. This result indicates that the protection provided to these sectors during the lockdown period–permission to remain open–prevented unemployment during that period. However, with the opening of the economy, this protection vanished, and they became more exposed and potentially even more affected by COVID-19 unemployment.

The robustness of the model was tested by including/excluding blocks of regional variables and identifying possible changes in other variables’ coefficients. We found that the COVID-19 intensity coefficient is sensitive to the inclusion/exclusion of some regional variables. Thus, we further investigated if this set of regional variables constitutes good or bad controls for the relation between regional COVID-19 intensity and the probability of an individual becoming unemployed after the COVID-19 outset. Following Cinelli et al. (2022), we concluded that these regional variables are good controls and, thus, should be included in the model to avoid having a biased estimator for the COVID-19 intensity variable. Finally, the AIC and BIC statistics clearly indicate that the model with all regional variables–including COVID-19 intensity–is preferable to those that exclude some blocks.

## Conclusion

In Portugal, the impact of COVID-19 on unemployment was very significant. The number of new registrants in the IEFP centers from March 2020 until February 2021 increased by approximately 22% compared with the homologous period (considering only individuals employed before). Moreover, if it were not for the buffer effect of extraordinary and temporary policies applied by the Portuguese government, such as the simplified layoff scheme, this rate would probably have been even higher. Remarkably, unemployment increase was very heterogeneous among NUTS III, with some regions presenting YOY rates closer to 40%, while others observed a decrease in the IEFP records. Herein, we sought to understand the effect of regional heterogeneity on the individual odds of unemployment after the COVID-19 outset, compared with the odds of being unemployed before the pandemic.

An extensive database was built using individuals that lost their jobs during the COVID-19 crisis (from March 2020 until February 2021) and during the homologous period (from March 2019 until February 2020), corresponding to more than 800,000 individuals. Besides including the entire population who lost their jobs in the 2 years of analysis (unusual among the COVID-19 literature), our data also has the strength of considering detailed personal and job information. Moreover, using the region and the occupation identifications, the original information provided by the IEFP was matched with information from other sources, allowing our final data to include three-dimension variables. Three logit models were used to estimate how the influence of a specific variable on the unemployment odds changed with the COVID-19 outset. Thus, we could evaluate which characteristics make individuals more vulnerable to COVID-19 unemployment and how the regional context of the labor market influences the individual’s employment outcome in the downturn generated by the COVID-19 pandemic.

The results indicate a significant territorial heterogeneity related to the effect of the COVID-19 crisis on unemployment. Moreover, the inherent regional context (defined mainly by structural characteristics) seems to impact individual vulnerability to COVID-19 unemployment, which persists after controlling for a large set of personal and job characteristics. This observation is a warning signal for policymakers, not only confined to the COVID-19 crisis, as those systemic regional determinants may indicate a slowdown in regional growth and delay stabilization if no specific action is taken. Moreover, considering that the COVID-19 crisis has caused heterogeneous effects between regions across the World, our study should also be relevant to (and applied to) other countries.

Individual human capital reinforcement (both general and specific to the job) proved vital in protecting individuals from this new crisis. In addition to this result, it was observed that individuals living in regions with higher human capital endowments are also less vulnerable. Both individual and regional effects justify the need for national and local policymakers to promote education and training programs that take into account the extensive rearrangement of employment between sectors and occupations, the effect of structural trends such as automation and digitalization and the emergence of novel work organization models originated by the pandemic. Preparing to match the workers’ qualifications and employers’ needs will require putting in place upskilling and reskilling programs, targeting specific economic structures and the needs of regional employers. Additionally, regional policymakers must also pay attention to the territorial readiness to catch new opportunities generated by accelerating new business models and technologies, such as increasing online education, using digital platforms and working remotely. These changes require direct support to education and training programs and factors (sometimes considered “too basic”) such as high-speed internet coverage and generalized accessibility to communication packs and hardware, which are not always available.

The statistical insignificance of the variable COVID-19 intensity and the statistical significance of the variable related to local mitigation measures may mean that unemployment is more related to “the cure” than the disease. In fact, the lockdown period implemented by the government, although essential to contain the spread of COVID-19 (Bourdin et al., 2021), was the period when the unemployment increased the most.

Our results also confirm “that best pre-crisis labor performing regions were more vulnerable during the crisis compared to the lagging regions” (Giannakis and Bruggeman 2017, 1401) and that individuals living in densely populated regions are now more exposed to unemployment. These results confirm the disruptive nature of this crisis, and it may be an important signal to policymakers that the labor market dynamics may not go back to “business as usual”, since previously identified labor market trends (e.g., reorganization of work, digitalization, automation) have been accelerated by the pandemic. Additionally, the economic lockdown, the closing of borders, the interdiction of international flights, and the fear of being infected with COVID-19 substantially affected regions highly dependent on international trade and tourism.

We provide additional insights into the literature by using individual and regional variables, distinguishing between internal and external effects. For example, both the human capital level and hospitality industry weight at the regional level influence individual vulnerability even after controlling for these variables at the individual level. Therefore, less-educated workers and workers not working in the hospitality industry are also affected (positively and negatively, respectively) by a regional labor market with higher levels of human capital and higher hospitality industry weight.

This study focuses on a short and medium-term timeframe, restricting the analysis to quantitative data that may influence the shock’s immediate impact on workers. Thus, a limitation of our study is that it cannot assess the long-term impact that the COVID-19 crisis might have on unemployment, which should be followed up in future studies. Additionally, institutional and political factors at the regional level, e.g., the propensity to develop collaborative innovation actions, gathering different agents of the regional ecosystem, and the political leadership’s capacity to design and implement place-based strategies/policies, should be considered.

## Notes

1. Information on the Portuguese Islands (Madeira and Azores) is not totally covered by the IEFP database and therefore it was not considered.

2. For each NUTS III: $Variation\;rate=\frac{Number\;of\;Unemployed\;in\;COVID-19\;period}{Number\;of\;Unemployed\;in\;pre-COVID-19\;period}-1$.

3. The introduction of this new variable produces no significant changes in the values and levels of significance of the remain coefficients, although it reduces the number of observations due to the existence of missing values.

## Appendices

**Appendix A.** Variable Descriptions and Summary Statistics.

Variable (1)		Description (2)	Pre-COVID mean (SD) (3)	COVID mean (SD) (4)
Personal characteristics	Gender (male)	Dummy: 1 if the individual is male; 0 otherwise	44.7% (0.5)	46.5% (0.5)
	Age	Age, in years, at the IEFP registration moment	39.5 (12.1)	38.9 (12.0)
	Higher education	Dummy: 1 if the individual detains a higher education diploma; 0 otherwise	15.9% (0.4)	13.5% (0.3)
	First unemployment	Dummy: 1 if the individual has never been unemployed before; 0 otherwise	17.4% (0.4)	20.1% (0.4)
Job characteristics	WFH feasibility	Variable that identifies the WFH feasibility of previous occupation (Martins 2020)	0.19 (0.3)	0.17 (0.3)
	Job-specific skills–level 2	Dummy: 1 if the occupation requires level 2/3/4 skills to be performed; 0 otherwise (INE 2011)	56.2% (0.5)	58.1% (0.5)
	Job-specific skills–level 3		8.0% (0.3)	7.7% (0.3)
	Job-specific skills–level 4		11.5% (0.3)	8.9% (0.3)
	Different occupation	Dummy: 1 if the individual is searching for a job in a different occupation than his/her last; 0 otherwise	34.9% (0.5)	32.6% (0.5)
	Temporary job	Dummy: 1 if the previous job of the individual was temporary; 0 otherwise	62.6% (0.5)	66.2% (0.5)
	Permanent job (involuntary disruption)	Dummy: 1 if the individual with a permanent work contract was dismissed from his/her previous job; 0 otherwise	15.3% (0.4)	18.2% (0.4)
	Permanent job (voluntary disruption)	Dummy: 1 if the individual with a permanent work contract voluntarily decided to leave his/her previous job; 0 otherwise	6.9% (0.3)	3.6% (0.2)
	Permanent job (mutual agreement)	Dummy: 1 if the individual with a permanent work contract agrees with the employer to end his/her job; 0 otherwise	5.0% (0.2)	4.3% (0.2)
	Self-employed	Dummy: 1 if the individual was previously self-employed; 0 otherwise	1.4% (0.1)	1.3% (0.1)
	Number of observations		366,779	447,151

Differences between the means of columns (3) and (4) are statistically significant for all variables (*p*-value <0.01) except for the self-employed variable.

**Appendix B.** Regional Variable Descriptions and Summary Statistics.

	Variable (1)	Description (2)	Mean (SD) (3)
Regional characteristics	Pre-crisis unemployment	Ratio of unemployed individuals to the residence region’s total population (2016–2018 average)	4.2% (0.9%)
	Population Density	Population density in the residence region of the individual (per km^2^, 2018)	483.4 (392.6)
	Human capital intensity	Average number of years of schooling in the regional labor market (2018)	10.3 (0.7)
	Exports of goods	Ratio of the value of exports of goods in total turnover (2016–2018 average)	15.1 (8.4)
	Employment in hospitality sector	Percentage of employment in accommodation and food services in the residence regional total employment (2016–2018 average)	9.3 (5.4)
	Employment in essential sectors	Percentage of employment in agriculture, forestry and fishing; electricity, gas and water activities; transportation and storage; public administration and defense; social security; education; human health and social work activities in the residence region total employment (2016–2018 average)	29.5 (8.2)
	COVID-19 intensity	Average number of COVID-19 cases per 10 thousand inhabitants in the residence region	50.1 (14.0)
Number of observations			801,798
